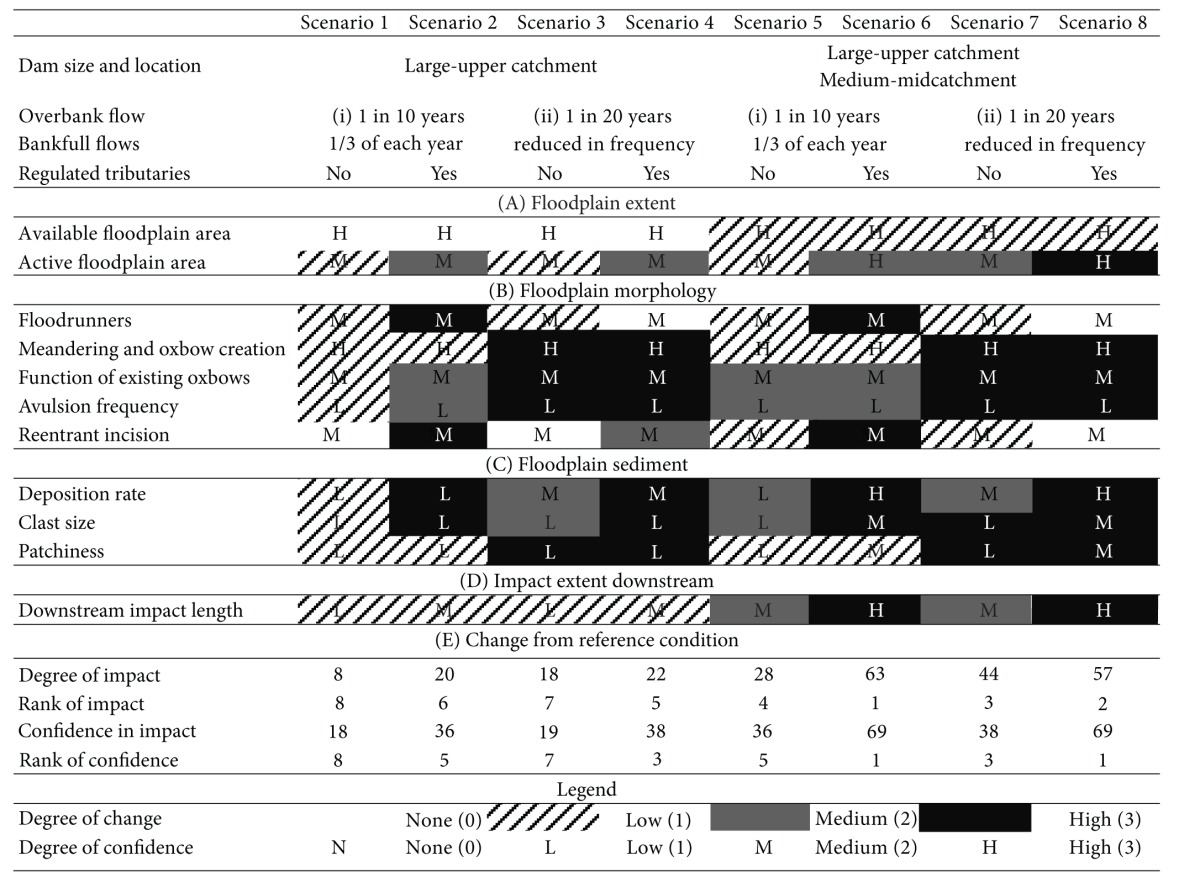# The Potential for Dams to Impact Lowland Meandering River Floodplain Geomorphology

**DOI:** 10.1155/2014/309673

**Published:** 2014-01-22

**Authors:** Philip M. Marren, James R. Grove, J. Angus Webb, Michael J. Stewardson

**Affiliations:** ^1^Department of Resource Management and Geography, The University of Melbourne, Parkville, VIC 3010, Australia; ^2^eWater Cooperative Research Centre, Australia; ^3^Department of Infrastructure Engineering, The University of Melbourne, Parkville, VIC 3010, Australia

## Abstract

The majority of the world's floodplains are dammed. Although some implications of dams for riverine ecology and for river channel morphology are well understood, there is less research on the impacts of dams on floodplain geomorphology. We review studies from dammed and undammed rivers and include influences on vertical and lateral accretion, meander migration and cutoff formation, avulsion, and interactions with floodplain vegetation. The results are synthesized into a conceptual model of the effects of dams on the major geomorphic influences on floodplain development. This model is used to assess the likely consequences of eight dam and flow regulation scenarios for floodplain geomorphology. Sediment starvation downstream of dams has perhaps the greatest potential to impact on floodplain development. Such effects will persist further downstream where tributary sediment inputs are relatively low and there is minimal buffering by alluvial sediment stores. We can identify several ways in which floodplains might potentially be affected by dams, with varying degrees of confidence, including a distinction between passive impacts (floodplain disconnection) and active impacts (changes in geomorphological processes and functioning). These active processes are likely to have more serious implications for floodplain function and emphasize both the need for future research and the need for an “environmental sediment regime” to operate alongside environmental flows.

## 1. Introduction

The riverine landscape is generally defined as a longitudinally continuous corridor consisting of those parts of the landscape directly affected by the river (i.e., channel, riparian zones, and floodplains). The floodplain has long been recognized as a significant part of the riverine landscape [1–3], and the importance of lateral connectivity between river channel and floodplain for ecological function has been repeatedly highlighted [4, 5]. Geomorphic processes affecting the formation of floodplains have also been well studied and indicate that floodplains are formed by a combination of relatively frequent within-channel or bankfull flows driving lateral channel migration and overbank flow processes driving vertical accretion and channel switching (e.g., [6–9]).

While the majority of these studies have examined processes in unregulated rivers, many of the world's lowland floodplain rivers are regulated by dams to some degree [10]. Globally, there are over 45,000 dams above 15 m high, affecting over half the world's large rivers [11]. Dams are recognized as an overarching impact on all aspects of river systems. One well-recognized impact of dams is a reduction in flood frequency and magnitude [12–15]. For example, in the United States, dams have reduced peak discharges by an average of 67% [16], and 55% of large rivers in the United States have had a greater than 25% reduction in the mean annual flood [17]. The importance of these events for ecological functioning of floodplains is well recognized (e.g., [18]). In contrast, despite recognition that much of the sediment deposited on floodplains is derived from peak flood events [19], the geomorphic implications for the floodplain of the reduction in flood frequency are poorly understood. Accordingly, although bankfull flows are often recommended in environmental flow assessments for the purpose of channel formation (e.g., [20]), overbank flows are recommended for ecological rather than geomorphological purposes (e.g., [21]).

It is often stated or assumed that the primary impact of dams on floodplains is in inhibiting or preventing overbank flows [22, 23]. Disconnection of the floodplain and the channel, with a cessation or reduction in the rates of floodplain geomorphological processes, would therefore seem to be the likeliest impact of dams on floodplains. The presence of flood control levees on many regulated floodplains also acts to compound this effect [24, 25] and it is often difficult to separate out the two impacts. Floodplain geomorphology is however usually considered to be the result of both vertical accretion and lateral accretion of the channel, with lateral accretion deposits forming as much as 80% of the total floodplain stratigraphy [6, 26]. Because of this, within-channel processes remain important in determining floodplain geomorphology, and rejuvenation of vegetation successions by channel migration plays a vital role in maintaining floodplain ecosystems [27, 28]. In order for the processes that maintain floodplains to be included in environmental flow assessments, we require increased understanding of the effects of dams on the within-channel and overbank processes affecting floodplain formation, and how these will be affected by dam-regulated flows. In this review, we draw on work from both dammed and undammed river systems to inform the development of a conceptual model of the likely effects of dams on floodplain evolution.

This review focuses on the impact of dams on medium to low energy lowland river meandering floodplains. This includes all of the floodplain types encompassed by the Types B3 and C1 of Nanson and Croke [29] and also some types of anabranching or anastomosing floodplain, where the individual anabranches are comprised of meandering channels formed of clastic sediment. It is acknowledged that this does not encompass either the full range of floodplain types, or the full range of floodplain types which have been affected by dams. However, this is justified in that meandering rivers form by far the greater part of the rivers across the globe [30], and as discussed below, these floodplain types are highly likely to be impacted by dams and flow regulation. Using this approach allows this paper to examine an interrelated set of floodplain processes.

## 2. A Framework for Consideration of Dam Impacts on Lowland River Morphology

### 2.1. The Downstream Continuum

Leopold and Maddock introduced the concept of the river as a downstream continuum, in which a range of geomorphic variables such as slope, flow velocity, and channel size adjust towards an equilibrium responding to downstream changes in discharge and sediment size [31]. Dams can be thought of as disrupting the downstream continuum [22, 31] by altering the primary geomorphic controls (i.e., flow and sediment regimes). The disruption is imposed upon a predictable downstream pattern in the sensitivity of rivers to altered flow and sediment regime. This disruption is a “press” disturbance in that once the disturbance is applied it remains in force. However, the intensity of this disturbance will be attenuated downstream as a consequence of unregulated tributary inflows [12]. The alignment between the intensity of geomorphic disruption and river sensitivity will determine the nature, rate, and extent of channel and floodplain changes.

**Figure 1:**
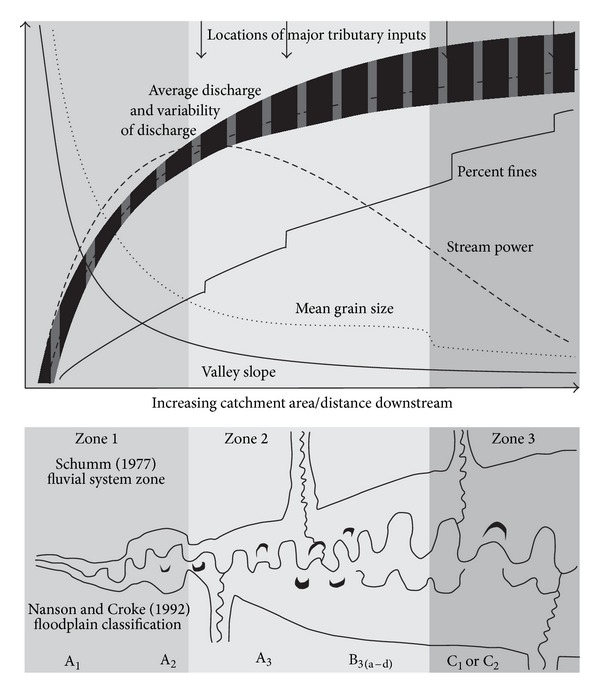
Longitudinal profile and downstream changes for an idealized catchment. The upper panel shows the downstream changes in slope, discharge, discharge variability, grain size, percentage of fines, and stream power expected in a catchment with a typical exponential long profile and a regular pattern of downstream tributary inputs. The lower panel shows the expected pattern of decreasing confinement in a downstream direction, with increasing meander wavelength, and a midcatchment peak in meander migration rates (and cutoff formation). The three zones correspond to the Schumm erosion, transport, and deposition zones [36]. The approximate locations of floodplain types according to the Nanson and Croke classification are also shown [29].

It is possible to generalize the longitudinal patterns in the sensitivity of rivers to altered flow and sediment regime. As drainage basin area increases, discharge generally increases and channel slopes decrease (Figure 1). Since stream power is related to the product of discharge and channel slope, these counteracting trends can produce a midcatchment peak in stream power [32], which depends on the arrangement of tributaries within the catchment [33]. The upstream sections of the catchment are frequently dominated by confined reaches [34, 35], direct contact of the channel with hillslopes, and coarse bed sediment or bedrock. Moving downstream, partly confined river sections result from a widening of the river valley, and coarse stored alluvium may be present in floodplain pockets. Either progressively or rapidly, the river halts contact with colluvium and becomes a laterally unconfined floodplain system (Figure 1). This is often associated with a change from an erosional regime to a transportation/storage one [36]. These downstream transitions are indicated as Zones 1 (erosion), 2 (transport), and 3 (deposition) on Figure 1. High stream powers combined with gravel-sand grain sizes mean that the system is highly reactive at this point, resulting in the development of a meander train [7, 29]. A rapid transition between sand and gravel in the bed [37] grades to more cohesive sediment at the lower end of the catchment [38]. Bank material also decreases in size fraction downstream but this is not such a clear trend compared to that of bed material [39]. The resultant efficacy of riverbank erosion processes may vary in the downstream direction alongside other trends, with mass failures only able to occur once the banks have become cohesive enough [40, 41].

### 2.2. Dams and the Downstream Continuum: Spatial Changes and Impacts

The impact of dams on the downstream continuum is dependent on where dams are located within the catchment and by the operational procedure they use. Inventories of dams are relatively common (e.g., [42]), and whilst they frequently contain hydrological information such as upstream catchment area they rarely contain explicit information on where the dams sit within the downstream continuum, or upstream catchment area relative to downstream. For small dams (<4 m high) geomorphological conditions may be similar upstream and downstream of the dam [43], but this is unlikely to be the case for large dams with the potential to significantly alter downstream hydrological conditions. In the absence of an existing discussion in the scientific literature on the typical locations of dams within a catchment, we examined a dataset of large dams located within southeast Australia in order to investigate the location of dams relative to confined and floodplain reaches.

**Figure 2:**
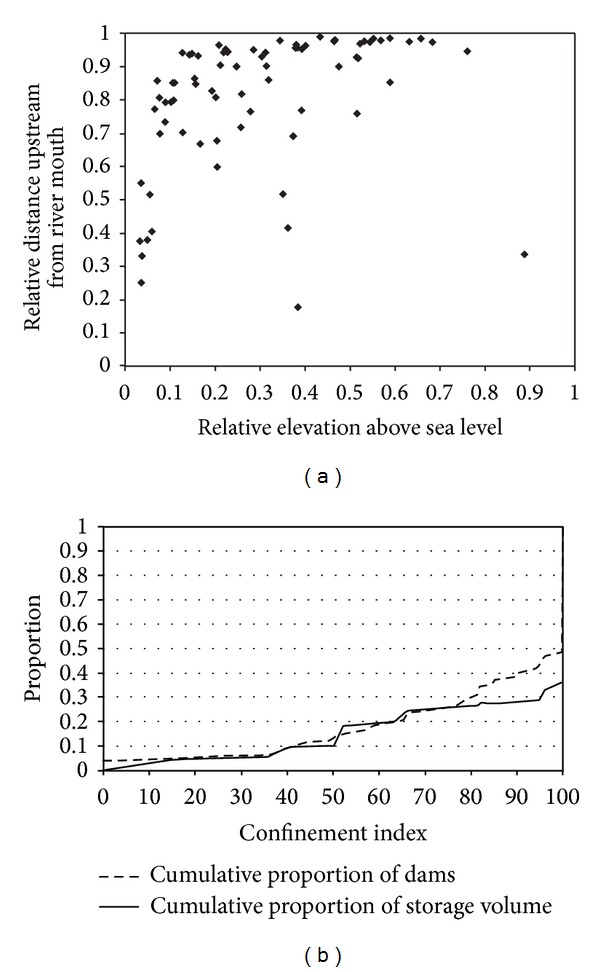
(a) Elevation and longitudinal position of dams in southeast Australian catchments (elevation is given as a proportion of the maximum elevation upstream of the dam and distance upstream of the river mouth is expressed relative to the maximum stream distance from the catchments headwaters to the river mouth). (b) Cumulative proportion of dam volume and dam count with increasing valley confinement in southeast Australia.

Analysis of 80 dams (see Appendix 1 in Supplementary Material available online at http://dx.doi.org/10.1155/2014/309673), all of which have catchments greater than 100 km^2^ and storage capacity greater than 20% of annual inflows, indicates that most are located in the upstream third of the longitudinal profile (65 dams) but at a height less than two-thirds of the maximum headwater elevation upstream of the dam (78 dams) (Figure 2(a)). An index of valley confinement was obtained for each dam site, derived from a modified version of the valley-bottom flatness index of Gallant and Dowling [44]. Approximately three-quarters of the total number of dams are located in confined valleys (confinement index greater than 80) representing 75% of the cumulative dam volume (Figure 2(b)). The historic data contained in Appendix 1 shows that for major river systems such as the Murray River dams were built first on the main stem and major tributaries, in “optimal” locations. In the Murray catchment, the earliest large dams were the Hume on the Murray itself in 1936 and Eildon dam on the Goulburn River in 1927. Dams were only built on smaller tributaries and in less optional locations, during the 1950s and 1960s. Together, these data indicate that in southeast Australia, large dams are typically located at the lowest practical elevation in confined valleys, placing them immediately upstream of floodplain reaches.

Dams operate for water supply, irrigation, hydroelectricity generation, navigation, flood control, or some combination of uses. The exact nature of the impact of a dam on the flow and sediment regime will depend on the size of the dam and the purpose it is used for. However, dam capacity relative to inflow volumes is a key determinant of the magnitude of downstream flow and sediment regime changes. The most highly regulated river systems can hold back more than an entire year's worth of discharge, although river systems where as little as one percent of the annual discharge is held back can also be strongly affected [11]. Larger dams relative to inflows generally lead to a greater reduction in flood volumes by retaining or delaying floodwaters [45, 46] and reducing suspended sediment loads through deposition within the dam [47]. Sediment flushing from the impoundment (and ultimately dam removal) will mitigate the effects of sediment trapping on downstream sediment loads. These effects are also attenuated further downstream by contributions of sediment and flow from unregulated tributaries [45, 48] or buffered by alluvial sediment stores [49].

The longitudinal pattern of geomorphic disruption produced by dams is superimposed on the downstream fluvial geomorphology continuum. The impacts can be thought of in terms of reductions in stream power caused by the downstream reductions in discharge, or more usefully in terms of the altered balance between stream power and the sediment load. This concept is expressed in the famous “Lane equation” [50], which considers channel change (specifically erosion and deposition) to be driven by changes in water (*Q*) and slope (*S*) relative to sediment load (*Q*_*s*_) and grain size (*D*):

(1) $$\begin{array}{c} Q_{s}D\propto QS. \end{array}$$

Changes in any of these parameters must inevitably lead to changes in the others, in order to maintain equilibrium. The Lane equation is qualitative, as it is not dimensionally balanced, but when rewritten as a balanced equation, it essentially becomes the Shields equation for sediment transport [51]:

(2) $$\begin{array}{c} \theta_{c}=\frac{\rho gdS}{g\left(\rho_{s}-\rho \right)D}, \end{array}$$

where *θ*_*c*_ is the critical entrainment threshold for a given grain size (*D*), *ρ* and *ρ*_*s*_ are the density of water and sediment, respectively, *g* is acceleration due to gravity, and *d* is channel depth. Pre- and postdam changes can be quantified using three metrics based on the Lane equation and the Shields equation [52]. The ratio of sediment supply and sediment capacity defines the sediment mass balance, whilst the Shields number defines competence, and hence incision potential and the ratio of the pre- to postdam two-year flood define flood magnitude and the capacity of a channel to maintain its predam channel width [52].

**Figure 3:**
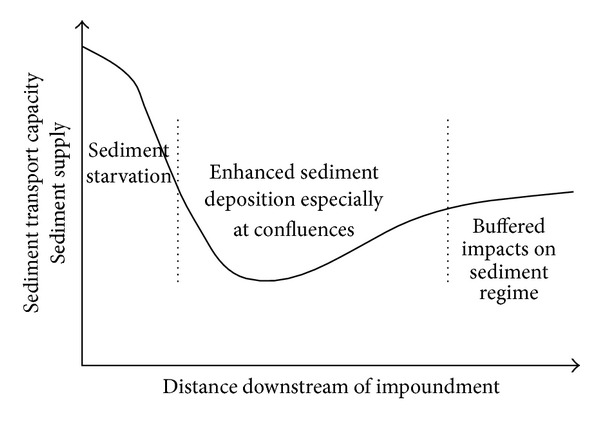
Conceptual model of the changes in sediment regimes downstream of dams. The *x*-axis represents distance downstream from the impoundment, with the area of sediment starvation beginning immediately downstream of the dam wall. *y*-axis represents sediment supply and transport capacity. Vertical dotted lines mark the transitions between downstream reaches dominated by different processes. Modified from [53] with permission.

Immediately downstream of the dam there is normally a reduction in both sediment load and flood magnitude (Figure 3), although total flow volumes may remain unchanged downstream of dams used for flood protection, hydropower, and irrigation storage. Sediment starvation is common in this zone leading to sediment transport capacity exceeding sediment supply. In alluvial reaches, channel bed and banks can be eroded leading to channel enlargement [45].

In unconfined lowland reaches the altered sediment regime is buffered by the intervening alluvial sediment stores [49, 54]. It is common for there to be some undammed tributaries, which deliver a natural flood regime to the lowland reaches. Impacts on sediment and flow regimes can be relatively small in this zone (Figure 3). The third zone only occurs in basins with significant lowland reaches. In coastal basins with short floodplain reaches, reduced flood volumes and sediment loads are likely to persist downstream to the estuary.

Where undammed tributaries provide significant sediment contribution, reduced sediment transport capacity typically results in deposition of sediments at confluences [45]. Where upland tributaries flow across the main floodplain before entering the dammed river, significant sediment deposits can also occur in the short floodplain reach of the tributary. These sediment stores will be either stabilized by vegetation or reworked in subsequent higher flow events in the main channel and redistributed downstream from the confluence. Extended interflood periods downstream of dams promote opportunities for vegetation colonization and stabilisation of sediment stores.

The location and contribution of dammed and undammed tributaries longitudinally along floodplain rivers controls the extent of each “disruption” zone. As discussed above, dam construction usually commences along the highest yielding tributaries in terms of flow, and dams are most commonly located at the lowest practical elevation in confined valleys, which offers the opportunity for construction of large capacity dams. For water supply, tributaries with greater water yield are most likely to be targeted, while hydroelectric power generation will require higher valley gradients (with the potential for higher sediment yields). The level of development and hence extent of flow and sediment regulation may vary from a single major dam, usually on the largest tributary entering at the upstream end of the floodplain, to damming of all the major of tributaries entering laterally along the floodplain. Under these conditions the zone of sediment starvation will extend further downstream from the head of the developed floodplain, as dams are located on a greater number of high yielding tributaries.

### 2.3. Dams and the Downstream Continuum: Temporal Changes and Impacts

The downstream impact of dams usually commences immediately following dam closure [12]. Bed degradation downstream of the dam is initially rapid but decreases over time. Typically, half of all degradation downstream of a dam will occur within the first 10 years [12]. Relaxation times for complete channel adjustment can however last several hundred years. There are no data to indicate the timescales over which dams will affect floodplain processes. A reasonable assessment of the impact of a dam on floodplain processes can be made by comparing the timescale over which dams operate, with the timescales of the fluvial processes that may be affected by changes in discharge and sediment regime. The life expectancy of dams is highly variable [55], although for large dams that are well maintained, life spans of 300 to 1000 years are feasible. Smaller, poorly maintained dams in areas with high sediment yields can have much lower life spans, ranging from 20 to 200 years [55].

Floodplain processes operating over timescales of decades to hundreds of years are therefore likely to be impacted by dams, whilst floodplain processes occurring over timescales of more than 1000 years are unlikely to be affected significantly by dams. This less than one-thousand-year threshold coincides with the “modern” time span in the threefold classification of Schumm and Lichty [56]. Short-term changes in water and sediment regime over the “present” time span (approximately 1 year) are likely to control the mechanisms that translate sediment transport processes into changes in floodplain landforms [57]. Simple models of cause and effect and of timescales of geomorphic processes are however complicated by the presence of geomorphic thresholds within fluvial systems [58, 59]. Where geomorphic change relies on some intrinsic threshold being crossed, dam construction may seem to have no impact if the floodplain was close to the threshold for change: the change may occur anyway. In other cases, dam construction may greatly reduce the likelihood of a threshold being crossed, thus extending the intervals between floodplain changes.

## 3. Floodplain Inundation

Lateral connection between river channels and floodplains is essential to maintaining floodplain wetland habitats [3]. The usual effect of flow modification, either through the modification of flow regimes by dams or by channelization and levee construction, is to modify, interrupt, or destroy this channel-floodplain linkage [22, 23, 60, 61]. Floodplain inundation extent and duration are the product of a range of water inputs and outputs [62]. The primary driver of floodplain inundation is the transfer of water from the main channel to the floodplain during periods of overbank flow. Surface water is lost from the floodplain by evaporation and return flow to the river from the floodplain, either by overland flow or by flow through the groundwater system following infiltration. Some water may also be lost to deeper groundwater systems. By reducing the frequency of overbank flooding, dams disrupt this process.

The phases of floodplain inundation by an overbank flood event are summarized in Table 1 [8, 63]. At a broad scale the extent of floodplain inundation is determined by regional hydrological inputs and outputs. At the site-specific scale the pattern of inundation as water moves from the channel to the floodplain is highly dependent on topography, which is a legacy of a complex history of geomorphological processes. Water will enter the floodplain via low-points in the channel banks, where the channel intercepts topographically low areas of the floodplain, adjacent abandoned channels, animal trails, or levee breaches. Topographic lows such as oxbows will be occupied early in the floodplain inundation process, followed by low-elevation floodplain areas. Complete floodplain inundation may only occur during the largest floods, but periods of partial inundation (“flow pulses”) may, however, be as important in maintaining habitat heterogeneity [64] and may be of great significance in maintaining highly regulated river systems. Partial inundation via anabranch connectivity may be important in maintaining the functioning of anastomosing river floodplains [65].

Sediment distribution along and across a floodplain is a consequence of localized geomorphological processes such as meander migration, point bar formation, avulsion, and overbank deposition. At the reach scale, even quite subtle changes in stream power primarily due to gradient changes can result in marked changes in sediment distribution within a floodplain [29, 67]. Consequently, regulation can also be expected to modify floodplain sediment distribution. The grain size of material available to form the floodplain is the product of regional and catchment-scale controls. The grain size of the sediment making up the floodplain is therefore primarily dependent on the location of the floodplain reach within the catchment, but this will be moderated by sediment trapping in the impoundments of dammed river systems.

The grain size of floodplain sediments plays two key roles in controlling the rate of water flow from the floodplain to the river. Firstly, fine-grained sediments can act to seal floodplain and palaeochannel/oxbow surfaces, preventing infiltration to the groundwater and encouraging ponding or surface runoff [68]. Where ponding occurs, the length of time for which standing water will remain on the floodplain will be largely controlled by the rate of evaporation, as water in clay-sealed oxbows behaves independently of regional groundwater behavior [68]. Sediment grain size also controls the hydraulic conductivity of the floodplain sediments and thus the rate of return flow of groundwater to the river [69, 70]. Any effects of regulation on sediment grain size distribution will ultimately translate to changes in the rates at which floodwaters are returned to the river.

## 4. Impacts of Dams on Floodplain Processes

In the sections below, we synthesize the literature on the likely effects of dams on the dominant processes involved in floodplain formation. Each section ends with a summary discussing how the evidence discussed informs development of our overall conceptual model (Section 6).

### 4.1. Overbank Flooding

Changes in the pattern of overbank sedimentation are caused by changes in the magnitude and frequency of overbank flooding. Because of the variety of uses for dams, the effect of dams on large floods varies between locations. The most comprehensive studies of the hydrological impact of dams are those by Williams and Wolman, Magilligan and Nislow, and Graf [12, 13, 16], Using pre- and postdam data, Williams and Wolman showed that for 29 large dams in the United States, average annual peak discharges decreased to 3 to 91 percent of their predam values, with an average reduction of 39 percent [12]. Magilligan and Nislow used data from 21 dams in the United States and found that average maximum daily discharge decreased at 20 of the 21 sites, by an average of 55 percent [13]. Graf used data from 36 large dams, also in the United States, but used upstream and downstream sites to make hydrological comparisons. These large dams reduced annual peak discharges by an average of 67% (with a maximum of 90%) [16].

**Figure 4:**
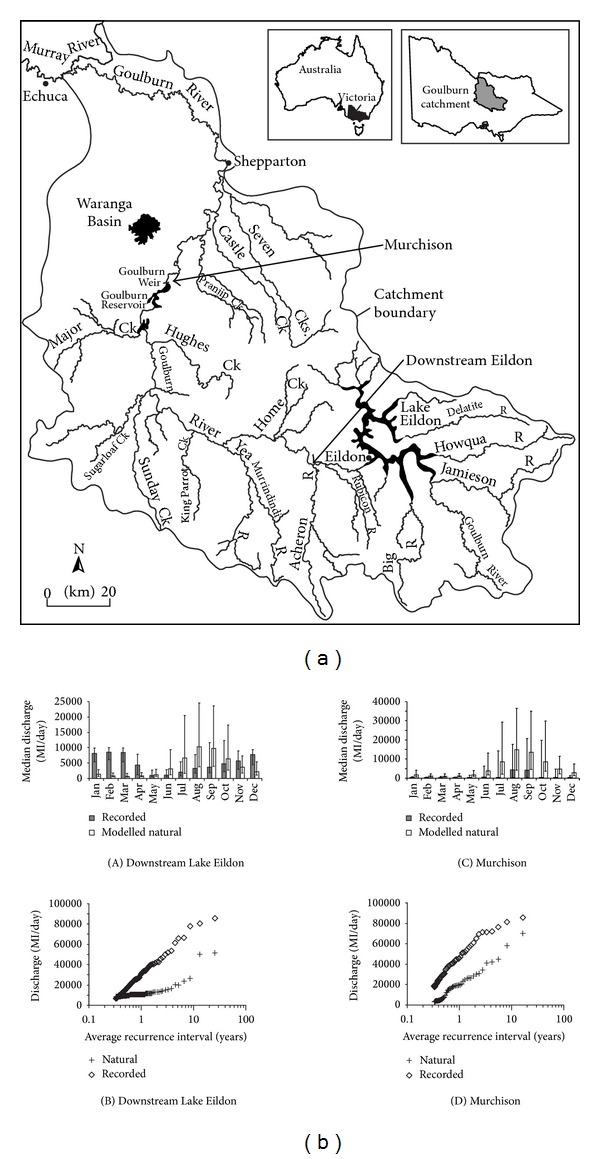
(a) Location map of the Goulburn River in Victoria, southeast Australia. (b) Recorded flows (under dammed conditions) compared with modelled natural flows for two locations on the Goulburn River. Eildon is immediately downstream of the major dam within the catchment. Murchinson is located midcatchment, downstream of Goulburn Weir, a major offtake and flow control structure. (a) and (c) are based on median monthly flows. (b) and (d) are flood frequency curves based on the partial series.

Outside of the United States, comprehensive regional studies of hydrological changes due to dams are less common, although there are many individual case studies. Higgs and Petts found that in the UK, mean annual floods on regulated rivers in the UK have reduced by approximately 30 percent following dam construction [71]. In Australia, most studies investigate the impact of dams within the Murray-Darling basin. These dams are frequently used for irrigation purposes, and so hydrological changes also vary in a downstream direction due to the effect of water abstractions. We use data from a tributary of the Murray, the Goulburn River, to illustrate this point (Figure 4(a)). Immediately downstream of the major water storage, Lake Eildon, flow regulation has the effect of reversing the natural flow seasonality and reducing the frequency of large flows whilst increasing the frequency of low flows (Figure 4(b)). At Murchison, downstream of the major irrigation offtake, flows retain their seasonality but are greatly reduced year round. At both sites, flows that would normally be expected to occur annually now have a 10-year recurrence interval.

Broadly similar trends occur on the Murray River itself. Immediately downstream of the Hume Dam, the dam has little effect on the size of average annual and peak discharges, although there is a pronounced reversal in flow seasonality [72]. Further downstream, the magnitude of flows which are exceeded 20 to 80 percent of the time is reduced by around 50 percent, although larger, rarer floods (recurrence intervals of 20 years or greater) are unaffected, because the irrigation dams have no capacity to store large volumes of floodwater [72]. This illustrates an important point about the impact of dams on overbank flows, namely that whilst most dams will reduce the size and frequency of moderate size floods, the very largest floods may still occur.

Our conceptual model is concerned with the impacts of dams over the 300–500-year lifetime of a “typical” large dam. The level of certainty regarding the effects of dams over this time period varies. In the short term (five years) we have considerable knowledge of the intended flow regime management strategy for a particular dam and can make predictions about how this will change overbank flows. Over an intermediate time period (50 years) we can have a high degree of statistical confidence that there will be increases in average (subbankfull) flows and a reduction in moderate to high flows, but the role of exceptionally rare large floods over this time period will be less certain. Over longer time scales (>500 years) we can be confident that there will have been one or more exceptional floods that were not moderated by the dam and resulted in significant overbank flows.

### 4.2. Overbank Sedimentation

Dams have the capacity to influence overbank sedimentation in two ways [73]. Firstly, the extent, duration, and timing of overbank flow will be altered under a regulated flow regime. Secondly, dams have the capacity to significantly alter the sediment load of a river, including the suspended sediment load that would otherwise be deposited on the floodplain. Dams are highly effective sediment traps, capable of trapping the entire sediment load entering at the upstream end of a reservoir [12, 74]. The effects of dams on floodplain reaches can however be mitigated in a number of ways. Changes caused by dams also need to be viewed in the wider context of catchment and land use changes. In many catchments, land clearing and agriculture increase fluvial sediment loads both prior to and concurrently to the construction of dams [75, 76]. Improved land use practices and the construction of dams may result in a decline in fluvial sediment to predisturbance loads, or lower, but trends are highly inconsistent because of the range of catchment development histories [76, 77].

As described above, undammed tributary inputs lead to a more natural flow and sediment regime developing downstream of the dam. However, in many highly dammed systems, the tributaries are also impounded, compounding changes to main stem channel sediment loads [78]. Channel scour and degradation take place immediately downstream of the dam [79] (Figure 3), increasing the sediment load from that point on. This latter effect is likely to have limited impact on the floodplain and will only occur during the period of channel adjustment after dam closure. Under dammed conditions, reduced flow magnitude, and increased frequency of subbankfull flows mean that most of the sediment scoured from the channel downstream of a dam will be redeposited within the channel, as it narrows in response to the new flow regime [80–82]. Thus, despite these factors acting to mitigate the effect of reservoir sediment trapping, suspended sediment loads have been shown to be only a small fraction of their natural load at distances of 100 to 500 km from a dam [12].

At a reach scale, floodplain aggradation is controlled by flood discharge and valley width [83, 84]. At the local scale, sedimentation on floodplains is controlled by the distance from the channel, the detailed microtopography of the floodplain, water depth, and grain size [85–87]. Sedimentation rates are greatest close to the channel, encouraging the formation of levee deposits [88]. The proportion of fine material increases with distance from the channel [89]. In standing water, sedimentation rates increase with water depth and are therefore greatest in abandoned and cutoff channels [90]. However the rate of infill is heavily dependent on channel orientation, with abandoned channels which have a high diverge angle from the man channel becoming isolated more quickly, and hence infilling with fine sediment rapidly compared to abandoned channel with a shallow diverge angle, which may stay open indefinitely [91]. Floodplain depressions therefore become increasingly important as locations of sediment deposition [85, 92]. Sediment transported across the floodplain may reenter the channel further downstream [93, 94].

During very high discharge floods, the floodplain may cease to act as a depositional location, as flow velocities can become high enough for sediment to remain entrained and for localized erosion to occur [94, 95]. The potential for floodplain scour is increased in dammed rivers where flows are unchanged but sediment loads are reduced [96, 97]. Floodplain sedimentation will also be reduced on unvegetated and cleared floodplains [98] and in situations where setback or single bank levees constrict the floodplain [99]. Both levee construction and vegetation removal will significantly affect sedimentation given that dam construction often accompanies floodplain development for agriculture (see also Section 5).

Dams have the potential for large-scale impacts on overbank sedimentation, although there are few studies that have documented changes in floodplain sedimentation following dam construction. The reduction in both overbank flow frequency and sediment load will combine to reduce levee and overbank sedimentation rates. Sediment accumulating on floodplains may become increasingly fine-grained when only the finest component of the suspended load passes through the dam [100]. Changes to floodplain sedimentation may be compounded by the fact that flow regulation often occurs in combination with the construction of artificial channel levees and floodplain earthworks [24, 25]. In a downstream direction, changes in overbank sedimentation will be large near the dam because of the reduction in sediment load and the reduction in peak floods. Because larger floods may still occur, there is also potential for floodplain scour, where overbank flooding occurs but upstream sediment is trapped. Further downstream, tributary inputs and erosion will diminish the effects of upstream trapping [101]. Downstream increases in bank height may encourage the persistence of channel and floodplain disconnection. Reductions in suspended load may become geomorphologically more significant downstream, where fine-grained sediment might have been expected to be deposited as part of the floodplain formation process [29]. Thus, although sediment trapping might be most striking near the dam, the geomorphological impact might be greater further downstream, where suspended load might have otherwise been expected to play a greater role in landform creation and modification.

The effect of reduced overbank sedimentation on levee development is dependent on the predam evolutionary history of the levee. Levee form varies with time since the initiation of levee development, and this may occur over hundreds to thousands of years [102]. Levee growth rates may reduce in the absence of significant suspended loads. Reductions in meander migration rates (see below) may partially counteract this effect, by maintaining levees in fixed locations for longer. Sedimentation on the inside of levees, by flows that do not overtop the banks, may contribute to channel narrowing. The overall pattern of change of levee sedimentation in response to dams is therefore complex and has not been studied in detail.

An altered ratio of lateral accretion to vertical accretion will, in the long term, alter the routing of surface water over the floodplain and groundwater through the floodplain. This will have implications for the duration of overbank flows. If oxbow lakes are infrequently inundated by flows with a low suspended load, they will not develop a clay seal and will not hold standing water for as long. Conversely, the rates at which oxbow lakes are infilled completely will decline, prolonging their longevity in the absence of sustained floodplain renewal by channel migration processes [103, 104]. Also, a long-term reduction in floodplain aggradation rate has the potential to alter the frequency with which floodplain renewal through avulsion occurs (see below).

For our conceptual model of floodplain impact, we can be confident that overbank sedimentation rates will be affected because of the high trap efficiency of most dams. Changes in overbank sedimentation are however also controlled by the pattern of overbank flooding discussed above. Thus, whilst it is reasonable to assume that trap efficiency and downstream variation in overbank sedimentation patterns will remain more or less constant over the lifetime of a dam, the actual changes in overbank sedimentation over time will be governed by the effects of regulation on overbank flooding patterns. So whilst there is a reasonable degree of certainty in the short term (five years) regarding the general tendency for reduced overbank sedimentation and increased within-channel sedimentation, the consequences of these changes are likely to be negligible. Over intermediate time scales (50 years) there should be a high degree of confidence that sedimentation patterns are changing and an increasing likelihood of these producing significant geomorphological changes. Over longer timescales (500 years) there is a greater likelihood of significant geomorphological changes because of reduced overbank sedimentation and increased within-channel sedimentation. Over longer timescales there are also likely to be floods that overtop the dam meaning there is an increased likelihood of sediment removal from both the channel and the floodplain and greater downstream variation in sedimentation patterns as a result.

### 4.3. Bank Erosion and Meander Migration and Cutoff

Continued channel change and migration via fluvial erosion and depositional processes act as a natural disturbance regime responsible for maintaining a high level of landscape diversity within the river corridor [3, 28]. Flood regimes in dammed rivers therefore need to be capable of maintaining continued channel migration.

Within-channel responses to dams alter downstream in response to changes in sediment loading [12]. Generally, the reduction in sediment load immediately downstream of the dam (Figure 3) increases the erosive capacity of flows. Enhanced bank wetting by low-medium flows maintained at constant levels also increases erosion [12]. Enhanced erosion, manifested as both bed scour and channel widening, usually occurs downstream of the dam, reducing in intensity further downstream. The downstream extent of bed scour varies greatly between dams [12]. Further downstream, reductions in discharge due to dams generally lead to reductions in bank erosion, often in association with channel narrowing. This reduction in bank erosion rates has the potential to alter rates of meander bend migration, with wider consequences for floodplain formation. On a meander bend in Wales newly deposited floodplain surfaces were up to 0.5 m lower following dam construction [105].

The formation of river meanders is an autogenic process, inherent in the river regime [106]. River meanders can change by migration, growth, and cutoffs [107, 108]. A number of early studies empirically related meander size to bankfull discharge [109, 110]. Adjustment of meander size should therefore be expected to follow changes in the discharge regime [107], and rates of channel change have also been shown to vary broadly in accordance with discharge regime, bank resistance, and stream power [111].

Some studies have shown a fairly direct relation between bankfull discharge and meander erosion. For instance, Hughes found that major erosional events that affected the full length of a meander were related to the 1.5-year recurrence interval events [112]. Analyses of this type ignore the importance of the downstream continuum described above and many studies show that the relation between discharge and bank erosion is not simple and linear [113, 114]. Bank erosion rates frequently peak in the middle reaches of a catchment [41], coinciding with the midcatchment peak in stream power rather than the downstream increase in discharge. The middle reaches may also be more prone to erosion because of downstream changes in grain size. Gravel-dominated upstream reaches and clay-dominated downstream reaches may be more difficult to erode than sand-dominated middle reaches. Relationships between discharge and erosion are further confounded by a range of other factors, particularly vegetation [115]. Consequently, Nanson and Hickin found that discharge or stream power can only explain ~45% of the variance in the rate of erosion of meander outer banks [116].

Channel migration is a discontinuous process, as the rate of cut bank erosion and the rate of point bar sediment accumulation at any one site are not necessarily in equilibrium [117–119]. The role of channel migration in creating floodplains has been demonstrated by recent modeling results [120] which show that abandoned and reactivated channels drive sediment and water distribution across the floodplain. The primary controls are the rate of channel migration and the rate of infilling of abandoned channels, which prevents them being reactivated [120]. Channel and floodplain geometry dictates that in rivers in steady-state equilibrium, more sediment is removed from the floodplain by bank erosion than is deposited in the channel on point bars [118]. The balance is made up of sediment deposited on the floodplain. If overbank sedimentation is reduced, there is likely to be a net loss of sediment from the floodplain. If both overbank sedimentation and bank erosion are reduced, overall rates of change will decline, but eroded sediment will be more likely to stay within the channel [121]. For the unregulated Little Missouri River, floodplain destruction is strongly positively correlated with the magnitude of infrequent floods, whilst floodplain formation is negatively correlated with the magnitude of low flows exceeded at all but the lowest discharges [119]. In the context of dams, the implication is that whilst the flows largely responsible for floodplain formation are less affected by dams, floodplain destruction rates will decrease. This will limit the availability of new space for floodplain formation and instead lead to increased within-channel deposition.

Supporting these hypotheses, a number of studies have shown that dams reduce meander migration rates [122–129] (Table 2). Conversely, discharge increases caused by water transfers have been associated with increases in meander migration rates [122, 130]. Meander migration models have also indicated that reductions in discharge will reduce reworking of floodplain material by meander migration [131].

In general, meander response to discharge changes is likely, but the magnitude of the response will be largely dependent on the nature of the bank material, antecedent conditions, and the nature of the basal endpoint control [114, 116, 133–135]. The short-term variations in response to variable magnitude flow events [113, 114] mean that even over medium time scales, there will not always be a direct relationship between discharge and bank erosion. For instance, after 20 years of monitoring, Hooke found that relationships between discharge and erosion established during one period of discharge pattern did not continue as discharge patterns changed, and other factors (vegetation in this case) had to be included to explain the variation in response [136]. In larger rivers, the effects of vegetation are less significant, and erosion rates may be more predictable [137]. In some dammed rivers, an increase in flows at the low to middle bank level has produced high erosion rates for considerable distances downstream of the dam [138, 139]. However, it is unclear whether these widespread increases in bank erosion were accompanied by a change in channel migration rate.

Nonetheless, over long time periods, relationships between discharge, bank material size, and meander migration rate seem relatively consistent. Thus, downstream changes, including sediment size and floodplain type [29] and in particular the average stream power of a reach, will play a large part in determining response to discharge variations, including those caused by dams. None of the studies describing the impact of dams on meander migration cited above assess whether the observed changes in migration rates showed any downstream variation. It is likely that based on the location of dams relative to the downstream stream power peak, changes in meander migration rate will be greatest in the middle reaches, relatively close to the dam, and that as discharge variations are buffered by downstream tributary inputs, changes in erosion and meander migration rates will reduce downstream.

Meander cutoffs and the formation of oxbow lakes are an integral part of the process of channel migration and are responsible for the creation and turnover of floodplain habitats and vegetation succession [140–142]. Oxbow lakes are also sites of preferential sedimentation during overbank flooding [8] and as such are sites of relatively rapid change in the floodplain environment, with several phases of deposition as the length of channel is abandoned [92]. Cutoffs trigger significant local erosion, which is usually deposited in the reach immediately downstream of the cutoff site, increasing channel complexity [143].

Meander cutoffs have conventionally been classified as neck or chute, with chute cutoffs generally more common than neck cutoffs [144, 145]. Chute cutoffs require periods of overbank flow in order to form, as the cutoff process involves a head cut linking the downstream side of a meander bend to the upstream end [146]. Neck cutoffs require only continuous meander migration in order to form [147, 148] but are usually completed by flood events [147]. Neck cutoffs are most frequent along sinuous, actively meandering rivers [145], and cutoffs help maintain average sinuosity in rivers with rapidly migrating meander bends [149]. Thus, meander cutoff formation is largely an inherent behavior of meandering rivers, independent of external variables [150, 151]. Boundary shear stresses sufficient for bank erosion to begin on meander bends at chute locations are dependent on channel curvature [152] and thus develop over time. Under this model, cutoff formation is clustered in space and time once a river reaches a threshold sinuosity and meander geometry [150, 153]. Modeling results indicate that river alternates between periods of meander extension and cutoff, with increased in-channel sedimentation occurring as the bend lengthens promoting overbank flow, which in turn triggers further deposition on the point bar. Thus, cessation of flow on the meander bend is accompanied by increased and concentrated overbank flow along swales, encouraging chute cutoff [154]. Flood events seem to be necessary to act as the final trigger for cutoff formation, but there appears to be no relationship between floods of a particular magnitude and cutoff formation [148, 150]. Instead, the cumulative period of overbank flow may be a better predictor [153].

Although it has been suggested that dams have the potential to affect cutoff formation rates [155, 156], there is little literature on postimpoundment impacts that demonstrates a change in meander cutoff formation rate. On the Sacramento River, cutoff rates have increased in the period following dam construction, but this is thought to be primarily due to clearing of riparian vegetation [153]. In most studies that implicate impoundments in the cessation of meander cutoff formation, dam construction appears to have almost completely inhibited meander migration (e.g., [125, 128]). In contrast, Wellmeyer et al. document a case where one cutoff occurred in a river in the 40 years prior to dam construction, but 2 cutoffs occurred in the 30 years following dam closure [129].

In principal, dams have the potential to affect the rate of meander cutoff formation. Continued migration and the development of excessively sinuous channels are an essential precursor to cutoff formation, and as discussed above there is a clear link between dam construction and reduced meander migration rates. However, the occurrence of cutoff formation is related to factors that are inherent in meander behavior, rather than external factors. Thus, while dams should have the capacity to delay the formation of cutoffs by slowing meander migration rate, it will not completely inhibit their formation unless meandering is stopped. Whether dams will have an immediate effect on cutoff formation rates will also depend on predam sinuosity and channel evolution history. It is likely that reductions in cutoff frequency will be greatest in the middle reaches of the river, where the potential for reduction in migration rates is greatest. There may be no change at all in the lower reaches.

Considering how this evidence contributes to the conceptual model, there appears to be a high probability that dams will result in reduced meander migration and meander cutoff rates. However, there is considerable uncertainty regarding the rates, timing, and direction of change compared to the likelihood of dams affecting overbank flow and sedimentation. Geomorphological responses to changing flow regimes are governed by complex feedbacks and thresholds that mean that predictable, direct responses do not always occur. In the short term (five years) the likelihood of meander migration rates changing is uncertain as there may be a lag as the river adjusts to the new flow regime. If the river is close to a threshold for meander cutoff formation, cutoffs that were “due” to occur may do so, irrespective of the change in flow regime. In the medium term (50 years), there is a greater probability that reduced meander migration rates will occur, and this may also result in a reduction in meander cutoffs, or the delay of “imminent” cutoffs. In the longer term (500 years) it is likely that occasional large floods will cause channel erosion. This may however take the form of catastrophic channel widening or incision, or floodplain stripping, in a similar fashion to that which occurs in alternating drought and flood dominated regimes [157]. Given that there are no long-term (500 years) observations of channel adjustments following dam construction, there is a great deal of uncertainty regarding the expected response.

### 4.4. Avulsion Frequency and Avulsion Type

Avulsions present an alternative mechanism for floodplain renewal and habitat generation. In particular, they create networks of abandoned or infrequently occupied channels that can act as water and sediment traps during periods of overbank flooding and hence form valuable habitat. Also, avulsions provide an opportunity for the establishment of pioneer species, as a new riparian habitat is created alongside the new channel. Avulsions occur over much longer time scales than channel migration and meander cutoff processes. Some studies (e.g., [128, 158]) have suggested that avulsions have ceased on formerly dynamic rivers following dam closure, but there has been no work to date that has demonstrated a direct link between dams and avulsion frequency. Nonetheless, there is some evidence to suggest that over longer time scales, dams have the capacity to change both the frequency and style of avulsions occurring on meandering river floodplains. There is also evidence that planned large releases from reservoirs have triggered avulsions in some rivers [125].

Avulsions are considered to be features of aggrading river systems [159], and avulsion frequency shows a positive correlation with aggradation rate [160–163]. However, superelevation of the channel is a necessary but not sufficient condition for avulsions to occur [164]. Factors favoring floodplain incision are also necessary [165]. The best known model of avulsion processes on alluvial floodplains is “avulsion by progradation,” where levee breaching and the development of crevasse splays encourage the gradual development of a new channel system by downstream progradation [159, 166]. In settings with high aggradation rates, progradational avulsions have frequencies of up to three per thousand years [167].

As discussed above, overbank aggradation is inhibited in many dammed rivers. In dammed rivers with an existing history of avulsions it is therefore likely that avulsions will decline in frequency. Where discharges are reduced, but tributary inputs increase sediment load downstream of the dam, channel aggradation can occur, potentially displacing flow from the channel and encouraging avulsion via floodplain scour (see below). However, because of the relatively short lifespan of dams compared to usual avulsion frequencies, it is unlikely that the presence of a dam could act to completely prevent avulsions from occurring.

Most floodplains with low sedimentation rates appear to be characterized by “avulsion by incision” [159, 162], and this mechanism may therefore apply on the floodplains of regulated rivers. There are relatively few descriptions of incisional avulsion in the literature, but in general, they rely on the occurrence of overbank flooding. Floodwater flows across the floodplain and eventually rejoins the main channel. Once connection with the main channel is made, upstream migration of a knickpoint creates a new channel [162, 168]. Incisional avulsions typically occur every several thousand years [159, 162] but develop relatively quickly over a period of a few years [169] up to 100–150 years [162]. Even where overbank flooding can occur, it is thought that the primary factor driving displacement of floodwaters from the existing channel into anabranches is the reduction in hydraulic efficiency in the main channel caused by increases in channel sinuosity over time [168, 170]. The reductions in meander migration rate discussed above will therefore also act to reduce the rate at which channels become “inefficient,” which may act to inhibit avulsion frequency in the long term.

With regard to development of the conceptual model, in the context of the timescales associated with impoundments (up to 500 to 1000 years), changes in avulsion frequency are only likely to be relevant in situations where a dam is located upstream of a rapidly aggrading, unstable floodplain. Given that a reduction in avulsion frequency involves slowing down an already slow process, it is unsurprising that no observed changes have been noted in the half-century of observations of floodplains affected by impoundments. In the short and medium term (5 to 50 years), dams are likely to have a negligible impact on avulsion processes. As avulsions are a threshold based phenomenon there is a possibility that large flow releases from dams may trigger an avulsion where it is “imminent” [125]. In the longer term (500 years) there remains the possibility that as sediment loads and aggradation rates are reduced on floodplains downstream of impoundments, there will be an increase in incisional avulsions associated with infrequent large floods causing floodplain scour.

## 5. Dams and the Effects of Vegetation on Floodplain Geomorphology

Dams have well-documented direct impacts on in-stream, riparian, and floodplain vegetation in regulated rivers (e.g., [60, 171–173]). There has also been substantial research on how floodplain geomorphology affects the distribution of floodplain vegetation, primarily via effects on local-scale moisture and oxygen (e.g., [174, 175]). For this review, we are interested in causal pathways in the opposite direction, that is, how dam-induced changes in vegetation may impact on floodplain geomorphology [176]. However, while the causal links between vegetation and channel form have been widely documented [120, 177], there is much less research on such effects on floodplains [178].

The establishment of riparian plants in rivers affects the physical processes shaping that river, thereby modifying patterns of erosion and deposition compared to what would have occurred otherwise. These physical modifications make the environment more suitable for further colonization by other species. Several studies have demonstrated changes in floodplain deposition and erosion rates following changes in vegetation (e.g., [179–181]), with some research going on to demonstrate that such changes affect the subsequent recruitment of floodplain vegetation [182]. Vegetation has also been recently implicated in enhancing or inhibiting chute cutoff frequency [152, 153]. Correnblit et al. referred to such an interaction of biological and physical processes as “biogeomorphic succession” and argued that rivers and floodplains could be considered as self-organising entities where interactions of biological and physical processes result in nonlinear trajectories and the possibility of multiple successional outcomes for seemingly similar systems [183, 184]. Importantly, the species involved have specific tolerances to different degrees of hydrologic variability, and beyond these thresholds the processes of biogeomorphic succession may break down. Ecosystem engineering of floodplains by vegetation thus requires repeated small floods to sustain habitat development. Perhaps as a consequence of this, while ecosystem engineering by plants has been observed across a wide variety of river types and climates, it has not been conclusively demonstrated in dammed rivers where these floods are lacking [184].

River damming may see different species being advantaged by altered flow regimes. For example, a reduction in floodplain inundation is expected to lead to an increase in the abundance of terrestrial-type species in areas that may have formerly supported inundation-tolerant taxa [185]. Such a change in species composition can be expected to lead to changes in the geomorphic impacts of vegetation. Therefore, even if damming does not completely close down the process of biogeomorphic succession on floodplains, it is likely to be affected to some extent by the direct impacts on vegetation.

The partial dependence of geomorphic processes on vegetation also means that floodplain development may also be affected by other influences on floodplain vegetation. For example, the invasion of exotic species into riparian zones is almost ubiquitous worldwide [186]. Some invasions are facilitated by dam construction, which can result in more favourable flow conditions for the invading species over endemic taxa (e.g., [187, 188]). These species will have different geomorphic impacts compared to the species they replaced and can thus be expected to differently affect channel and floodplain development. For example, *Salix* willow species have invaded many waterways in Australia. They have a competitive advantage over local species due to the greatly reduced variation in river levels that occur under regulation [189]. The root masses of these trees reduce channel cross-sectional area, which can increase flooding frequencies and promote channel avulsion [190], directly affecting floodplain development. Floodplains are also very commonly developed for agriculture (e.g., [138, 191, 192]). The resultant change in vegetation assemblages from mixed forest assemblages to cropping species could be expected to reduce the impedance of floodwaters by vegetation [193], potentially leading to greater shear stresses on floodplain soils. The sheer scale of these types of changes in floodplain vegetation means that they are likely to have far greater impacts upon floodplain development than any changes in vegetation directly induced by the dam itself.

Thus there are several potential links between dams, floodplain vegetation, and floodplain development. However, these influences remain speculative or have only been demonstrated in isolated case studies. The complexity of these systems, the presence of feedback loops and the spatial and temporal scales over which changes occur mean that identifying cause and effect relationships remain difficult [194]. Moreover, the scale of such effects compared to those induced by other influences on floodplain vegetation is likely to be small.

## 6. Discussion

### 6.1. Summary: A Conceptual Model of Dam—Floodplain Linkages

There have been relatively few studies that have specifically investigated the impact of dams on floodplain geomorphology. However, as indicated by the review above there is potentially an identifiable response which will be more complex than a mere cessation of activity. Despite knowledge gaps, we have shown that that understanding of floodplain processes is sufficiently advanced that the likely response to damming scenarios can be predicted, at least in a general sense.

**Figure 5:**
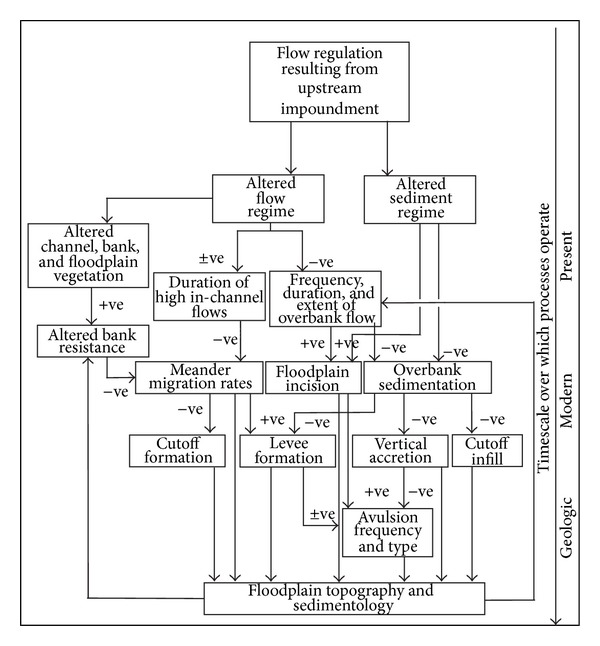
Conceptual model of the effects of dams on floodplain geomorphology. Timescales over which processes operate are arranged vertically through the model. +ve and –ve symbols indicate whether the changes induced by damming typically result in an increase or decrease in the rates of a particular process. Diagram is from Grove et al. [195].

Using the combination of observations from dammed rivers and knowledge of floodplain processes, we can develop a conceptual model of the chain of causes, effects, and feedback loops that will occur on a floodplain downstream of an impoundment (Figure 5). Necessarily, this entails some generalization, but it provides a series of linkages that are either well established or remain as hypotheses for further testing. Also, it indicates the timescales over which processes will operate and therefore the likelihood that disrupting a process will impact upon a floodplain during the design life of a typical dam. To maintain a closer link to the primary literature upon which it is based, the inputs to the conceptual model are spelled out within each of the final paragraphs of the subsections in Section 4. Here we provide a very brief recapitulation of the established and hypothesized causal pathways without further explicit reference to that literature. In general, our confidence in the linkages decreases moving from process operating in the “present” (top of Figure 5) to those operating over “modern” timescales and decreases still further for processes operating at “geologic” timescales (bottom of Figure 5).

The two primary drivers of change to fluvial systems in response to dams are changes in the flow regime and changes in the sediment regime. These changes are well established in the primary literature on dam impacts. The nature of the altered flow regime is difficult to generalize, as every dam is managed differently. Broadly though, there is likely to be a reduction in the magnitude and frequency of medium to high flows, and the variability of low flows is likely to be reduced, and either lowered, or seasonally raised where water is released for irrigation purposes. The nature of the altered sediment regime is easier to generalize, since most dams are efficient sediment traps across the full range of grain sizes. Flow and sediment regime modifications occur over short “present” timescales but have geomorphic impacts that extend over tens to hundreds of years (“modern” timescales).

Altered flow regimes impact upon both within-channel flows, where their greatest geomorphic effect is on bank erosion and meander migration rates and overbank flows, where the greatest impact is on the frequency and extent of overbank flooding. The main effects of altered sediment regimes are on the suspended load and hence overbank sedimentation. In the context of floodplain impacts, reductions in bedload are less important, except where they enhance rates of bank erosion and migration. Several studies of dam impact have reported a reduction in meander migration rates, and it is likely that this reduction will be accompanied by channel narrowing and vegetation establishment. Reduced meander migration rates may inhibit cutoff formation. Meander migration and cutoff formation operate on a decadal (“modern”) time scale, and hence effects are likely to be noticeable within 50 to 100 years of dam construction.

Reduction in overbank sedimentation is likely to reduce floodplain vertical accretion rates, although this has not been widely documented to date. Our understanding of floodplain processes indicates that this will inhibit levee growth, decrease topographic variation across the floodplain, and prevent the infilling of cutoffs and abandoned channels. Although a reduction in the rate of infilling of floodplain depressions will allow them to function as wetland habitats for longer, it will also inhibit the development of a clay seal at the base of the depression, preventing them from retaining standing water for extended periods. Overbank flooding with reduced sediment loads may encourage floodplain scour and reentrant scour (incipient head cuts) where floodwaters rejoin the main channel. Reduced vertical accretion rates may also lead to a reduction in avulsion frequency in highly dynamic floodplains. If avulsions do occur, they may also develop a tendency to develop by incision of the floodplain from headward cutting channels, rather than by downward propagation. The very long timescales over which avulsions occur, however, mean that impacts of dams on avulsion frequency are likely to be minor and there is a great deal of uncertainty regarding the relationship between dams and avulsions.

Possibly the biggest unknown, regarding the impact of dams on floodplains, is whether there will be a change in the rate of lateral accretion relative to vertical accretion. It is also possible that the rates at which these two groups of processes adjust vary in a downstream direction, so that their relative importance changes down the floodplain. Such changes would lead to alterations of floodplain character and topography as a direct consequence of damming. Floodplain construction is a consequence of events that occur on a subannual scale (flooding and bank erosion and deposition) producing geomorphic changes which occur over decades to hundreds of years. As such, it is reasonable to expect that large dams might have the capacity to modify floodplain character in this way. The long-term implication is that not only does damming lead to a reduction in the frequency, extent, and duration of overbank flows, but that geomorphological feedback mechanisms may result in changes to the floodplain that further alter the extent and duration of overbank flooding.

### 6.2. Scenarios of Changes to the Downstream Continuum

What changes do the linkages identified in this review produce in the floodplain downstream of an impoundment? There are too few observations of dam-affected floodplains to answer this question based on data alone, or with any degree of certainty. Instead, we can integrate the linkages between damming and floodplain processes (Figure 5), with the idealized downstream continuum (Figure 1) in order to make speculative predictions about likely geomorphological outcomes. All of these predictions involve observable and measureable changes to processes or rates of processes and are thus testable hypotheses, which await further testing.

Our analysis of dam locations indicated that most dams are in the upper third of the catchment, immediately upstream of the transition from confined to partly confined conditions. A second potential location inundates the floodplain itself, in the middle third of the catchment. Dams in this location are smaller and less common, but their impact can be significant due to their location and because they are often used specifically to divert water away from the floodplain. We consider two dam location scenarios: (1) a single large dam in the upper third of the catchment; (2) two dams in series, a large dam as above and a smaller dam in the midcatchment.

Alongside these two location scenarios, we consider two altered hydrological regimes: (1) a scenario in which flows are maintained at near bankfull for approximately one-third of the year, and all overbank flows are eliminated except those with recurrence intervals greater than 10 years (a hypothetical irrigation release dam); (2) a scenario in which flows with average (equivalent to bankfull) discharges are reduced in magnitude, and all overbank flows are eliminated except those with recurrence intervals greater than 20 years (a hypothetical water diversion dam). The dams are considered to be 100% effective sediment traps in both scenarios. Finally, we consider two scenarios related to tributaries, one in which all tributaries are undammed and one in which all tributaries are also dammed. We use a combination of these scenarios to give a total of eight possible changes to the downstream continuum.

These scenarios have the potential to lead to a diverse range and intensity of effects on floodplain geomorphology.  Table 3 outlines the predicted impacts of the eight scenarios, with change and intensity of change expressed as a departure from reference condition. Although each of the floodplain features or processes in Table 3 has the potential to be impacted by dams, there is insufficient information to weight the relative importance of change for each type of floodplain feature, so all are assumed to have equal importance. Also, change from reference condition means a change in a process or the rate of a process, which may lead to a change in form. It does not in itself imply that the floodplain is changing in ecological condition or is under any additional “threat.”

For the identified floodplain features, the degree of change from reference has been scored from no change (0) to high degree of modification (3) for each of the scenarios. Since changes in floodplain extent, morphology, and sediment are equally weighted; they are summed and then weighted by the distance downstream over which the impact is likely to extend. This produces a score for each scenario. The scores are ranked to provide an indication of which scenarios are most likely to move a floodplain away from its predam reference condition. The score is heavily dependent on our current understanding of geomorphological features that could potentially be modified by dams. Identification of additional features with different degrees of change from reference could produce a different set of outcomes from those shown in Table 3. Nonetheless, Table 3 provides a useful indication of the way in which a number of typical damming scenarios may affect floodplains.

We also evaluate our confidence in our scenarios, using a similar methodology to that used to assess impact. Our understanding of a particular process and of the available science underpinning our selection of its response to damming is judged on a high (3), medium (2), and low (1) scale. The summed confidence score is multiplied by the downstream impact confidence score to produce an assessment of the confidence of our scenarios. The confidence rankings are based on (1) how much is known about the process? This means that most “processes” get the same confidence rating irrespective of scenario as not enough is known enough about the processes to think about how they will be different with subtle changes in flood interval or bankfull flows; (2) how likely are they to be affected by multiple dams on the main stem or tributaries? Whilst more research needs to be done on how far downstream the floodplain will be impacted downstream of a single dam, we can be more confident that the floodplain will be impacted more significantly where there are more dams. Correspondingly, the downstream impact confidence score for some processes has been increased for the scenarios with multiple dams. Generally, degree of impact and degree of confidence follow similar patterns, although this is because both are driven more by the number of dams in the catchment and the downstream impact/confidence score than they are by our understanding of the processes, further highlighting the need for additional research on the links between dams and floodplain geomorphological processes.

**Figure 6:**
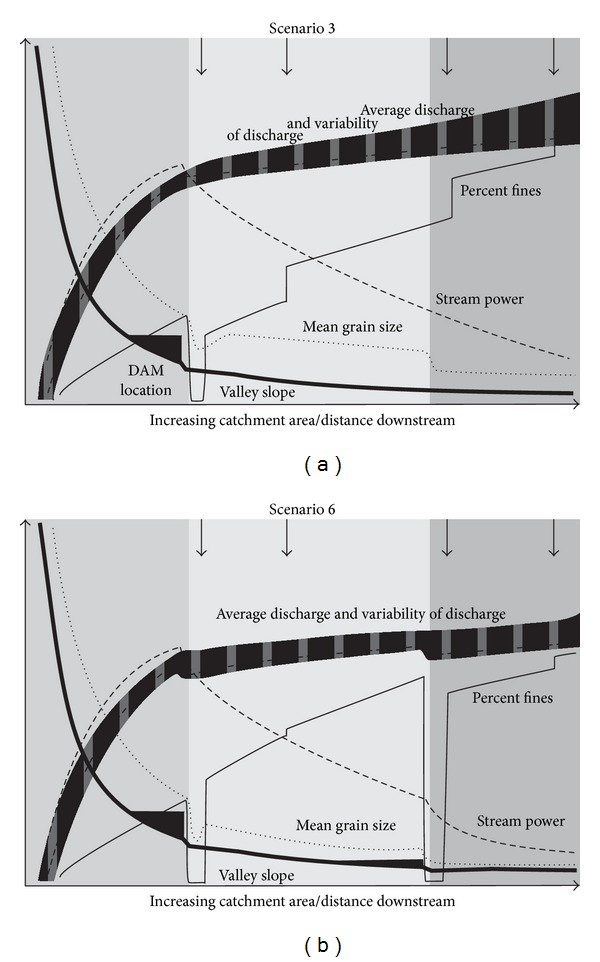
Potential impacts of dams on the downstream continuum, illustrated by modifying Figure 1. The two scenarios modelled correspond to Scenario 3 and Scenario 6 in Table 3. (a) One dam in the upper third of the catchment, with undammed tributaries and a hydrological regime where bankfull flows are reduced in frequency, and all floods less than 1 in 20 years are eliminated. (b) Two dams; one located in the upper third, as in A, and a second dam on the floodplain. In this scenario, the dams modify the hydrological regime such that flows are held at near bankfull for approximately one-third of the year, and all floods less than 1 in 10 years are eliminated. Tributary inputs are also regulated by dams.

The scenarios in Table 3 indicate a range of modification in response to damming. We will use two of the scenarios to explore the mechanisms for change in more detail.  Figure 6 shows changes to the downstream continuum introduced by cases 3 and 6. These scenarios have been chosen because they exemplify the range of dam effects.

In scenario 3 downstream impacts are limited (Figure 6(a)). This is because sediment starvation downstream of the dam, particularly the percentage of fine material, is quickly mitigated by the undammed tributary inputs further downstream. Construction for water supply means that discharge and stream power are reduced and there are consequent reductions in meander migration and cutoff rates. However, the minimal changes in the sediment regime mean that changes in floodplain sediment character are small. Changes to the flood regime are mitigated downstream by flooding from the undammed tributaries. Overall, the changes induced by this scenario have a moderate to high potential to induce change on the floodplain, but these changes do not extend very far downstream, producing an overall low impact. Similarly, given the lack of case studies which have documented long-term changes of this nature and our uncertainty regarding the extent to which the processes will be altered, we have an overall low degree of confidence in this scenario.

The hydrological regime changes in scenario 6 (Figure 6(b)) are less severe than those in scenario 3, with more overbank flooding. However, the combined effect of tributary damming and the blocking of the sediment continuum in two places produce greater overall effects, and the recovery of discharge and percentage fines back to reference is limited. A strong reduction in sediment load combined with a hydrological regime that still includes occasional overbank flows introduces the potential for increased floodplain scour and erosion of reentrants as floodwaters drain from the floodplain back into the channel. Dammed tributaries mean that the flood regime remains similar down the full length of the catchment. Stream power is reduced by the effects of two dams in series, and the presence of the second dam reduces the area of active floodplain due to permanent inundation of its reservoir area. Under this scenario, changes to the floodplain occur down the full length of the catchment, producing an overall high impact. This scenario also produces a high confidence score, but this is largely due to our confidence in the extent of impacts due to the presence of multiple dams on the main stem and tributaries. As with scenario 3, our confidence in the process alteration is limited by the lack of relevant case studies and the current incomplete state of knowledge regarding the processes concerned.

The above analysis identifies two types of “severe” impact. In scenarios such as 3 and 8, the impact on the floodplain is severe because it is isolated from the channel and because floodplain renewal has also slowed down or ceased. This is due to a reduction in the frequency in overbank flood events, along with a reduction in bankfull “channel forming” flows. Such changes in flow regime may occur because of damming and can be thought of as a passive response as the floodplain essentially becomes inactive. This scenario has obvious negative ecological expectations. However, in Table 3, the worst scenario, in terms of a shift from the geomorphological predam reference condition, is actually scenario 6. This is primarily due to an increased possibility of floodplain scour and erosion of reentrant channels. Also, overbank flooding with a low suspended load leads to reduced infill of oxbows and potentially the absence of a clay sealing layer on the bed of cutoff channels. This will reduce the extent of standing water on the floodplain and inhibit wetland habitat development. In situations where dams trap sediment on both the main channel and major tributaries but where dams allows overbank flooding (e.g., as environmental flow releases), the changed sediment regime introduces the possibility of large changes away from geomorphological reference conditions. Such changes can be considered active rather than passive. This observation emphasizes the difference between ecological perspectives on flow regulation on floodplain impacts (where hydrological changes are the main focus) and a geomorphological perspective (where the water *and* sediment regime must be considered). As we observed at the beginning of this review, floodplain geomorphology has not been considered in environmental flow assessments. Our discussion of Scenario 6 suggests this may be a serious omission.

### 6.3. Future Research Directions

In our conceptual model (Figure 5) and scenarios of dam impact (Figure 6 and Table 3) above, we indicate that the overall degree of confidence in the way in which dams will impact on floodplain geomorphological processes is still relatively low. This is particularly so for processes which operate over medium to long (“modern” and “geological”) timescales and for the way in which processes change in a downstream direction. There is an ongoing need to monitor and document the impacts of dams on river systems and for these studies to focus on the wider riverine landscape beyond the river channel and include hydrological, ecological, and geomorphological impacts.

The primary drivers of channel and floodplain change are lateral and vertical accretion and floodplain incision. There is a need for these processes to be investigated together, as the largest uncertainties regard the relative change in lateral to vertical accretion rates, and the controls on the interrelationship between floodplain deposition and floodplain scour. The way in which in channel and floodplain erosion and deposition change under altered sediment and hydrological regimes ultimately drives all of the processes discussed in this review, including channel migration rates, cutoff processes, overbank sedimentation and scour, levee formation, and avulsion frequency. There is a pressing need to not just study the change in rates of these processes individually, but to study the change in rates relative to the other processes. The role of vegetation in altering or moderating these processes is also currently very poorly understood and needs to be integrated more closely into studies of floodplain water and sediment dynamics.

These issues can be addressed via long-term monitoring projects, which should be instigated once dam construction is completed. For dams which are already in place, reconstructions of historic channel morphology, measurement of floodplain deposition rates, and sediment budget studies, using radionuclide tracers will provide new insights into how floodplain processes respond to upstream dam construction. Such studies will need to investigate dammed catchments and also undammed ones or be able to reconstruct both predam and postdam conditions. Given the variability inherent in riverine landscapes and dam construction scenarios it is unlikely that any one case study will fully explain or account for the range of potential dam impacts. This uncertainty will be partly dealt with as more case studies become available. Semiquantitative “weight of evidence” systematic reviews may provide a means of combining the evidence from multiple studies into one coherent argument, even where the individual lines of evidence are statistically weak [195], an approach which is relatively new in the discipline of geomorphology.

## 7. Conclusions

Investigations into the geomorphological impacts of dams and flow regulation have previously focused on changes to the channel. Studies of the impact of dams on the floodplain have primarily had an ecological focus. This review has attempted to address this knowledge gap by drawing together literature from both dammed and undammed rivers to assess the potential for dams to impact on the geomorphology of floodplains. There have been relatively few studies that have directly addressed the question of changes to the floodplain in response to damming. Nonetheless, we have identified a range of potential impacts on the floodplains of regulated rivers, many of which have not been identified or investigated previously. These impacts relate to changes in overbank and cutoff sedimentation, meander migration and meander cutoff rates, floodplain scour and incision, and avulsion frequency and changes in vegetation that may impact on geomorphology. In addition, most of these changes produce a feedback through changes in floodplain morphology and sedimentology, further altering the timing and distribution of overbank flooding. In our model and scenario we discuss the relative degree of confidence in the likely extent of process alteration and suggest that greater understanding is needed, particularly around the relative roles of lateral and vertical accretion and floodplain deposition and scour and the role of vegetation. Hopefully, the issues and questions raised in this review will act as a catalyst for future floodplain research.

Our review has enabled us to develop a conceptual model of the influence of dams on floodplain geomorphology and to investigate the possible effects of a number of flow regulation scenarios. The scenarios emphasize the range of changes from predam conditions that may occur. Significantly, we identify two types of impact: passive impacts and active impacts. In passive impacts, the floodplain essentially becomes an inactive terrace surface. In active impacts, changes in the sediment:water ratio delivered to the floodplain induce changes in geomorphological processes, such as enhanced scour and the absence of clay sealing layers in oxbows, preventing habitat development in abandoned cutoffs. From a geomorphological perspective, active changes are potentially more serious than passive changes, although it remains to be seen whether such changes will lead to differences in the ecological functioning of a floodplain. Even so, environmental flow assessments [195] in many regulated systems will need to incorporate an “environmental sediment” requirement if floodplains are to maintain their predam geomorphological functioning.

## Supplementary Material

A database of large dams in southeast Australia. ‘Large' is defined as having a catchment area greater than 100 km^2^, and a capacity capable of storing greater than 20% of average annual inflow. Some dams appear twice, indicating their original and enlarged capacities. The dams are ranked by catchment area. The ‘Purpose' column indicates the primary function(s) of the dams, using the key: (S) Storage for water supply; (I) Irrigation; (H) Hydropower; (F) Flood mitigation; (R) Stormwater retention; (P) Supply to coal-fired power stations.

## Figures and Tables

**Table 1:** Phases of floodplain inundation and drainage as suggested by various sources, modified from [8].

Source	Flood phase										
Allen (1970) [66]	Spilling of flood water from the main channel into empty flood-basins				Filling up of flood basins to a stage where sustained flow down the floodplain is possible				Emptying of flood basins		Drying out of flood basins and modification of newly deposited sediment

Lewin and Hughes (1980) [63]	Low water channel	Unvegetated bars and secondary channels	Groundwater rise and areas directly connected to channel via bank breaches	Higher parts of bars and breaches feeding more low relief areas	Bankfull promotes more rapid filling by overbank spilling and slows down the rate of stage rise in the channel	Internal transfer processes extend area of inundation	Floodplain filling: ponds begin to deepen rather than extend	Whole valley flooded: further increments lead to higher flow velocities and depths	River stage falling allowing overbank returns	Once below bankfull rate of recession depends on the efficiency of transfer processes and ebb channels to empty floodplain	Isolated disconnected ponds left in topographic lows to dry by infiltration and evaporation

Zwoli$\overset{´}{\text{n}}$ski (1992) [8]	Channel and groundwaters rise: erosive modification of floodplain edges (bank erosion)		Inundation of the floodplain: erosion and redeposition of older sediments; accretion of bars and levees		Adjustments of the overbank flow pattern to floodplain environment (morphology, vegetation, etc); transport dominant, but accretion occurs across floodplain			Flood peak: erosion declining, widespread transport	Initial fall of floodwaters: changes in overbank flow pattern; reduction in erosion and transport; peak deposition	Gradual cessation of floodwaters; transport ceases; final deposition and erosive modification of new deposits	Loss of stagnant water from depressions Postflood subaerial transformation of floodplain

**Table 2:** Migration rates before and after damming from five rivers in the USA indicating suppressed lateral migration rates following regulation. Data from [129].

Pre (m/year)	Post (m/year)	Transition (m/year)	Decreased migration (%)	River	Source
4.95	2.85	3.6	42	Trinity	[129]
1.75	0.45	3.4	74	Milk	[122]
3.4	1.8	N/A	47	Brazos	[132]
6.6	1.8	3.7	73	Missouri	[127]
5.6	1.3	4.3	77	Missouri	[123]

**Table 3:** Floodplain alterations from reference condition based on eight scenarios with the following variables: (1) 1 upstream dam; (2) 1 upstream and one midcatchment dam; (3) irrigation flows; (4) water supply flows; (5) dammed tributaries; (6) undammed tributaries. (A) The available floodplain area may be reduced by reservoir inundation, whilst the active floodplain will be reduced by lowering the area of flood extent. (B) Morphology may be altered by changes in the equilibrium between deposition and erosion. (C) The sedimentology may vary in rate, size, and also patchiness (or homogeneity) in both the cross and longitudinal profile of the floodplain. (D) The impact length downstream is used as a multiplier for (E) to weight the sum of A + B + C and then ranked from 1 (highest) to 8 (lowest) to denote modification from reference. The degree of confidence of each impact is rated None, Low, Medium, and High, based on the scientific understanding of the processes involved and also the cumulative impact of multiple dams, on both the main-stem and tributaries.