# Supplementary material for: The Potential for Dams to Impact Lowland Meandering River Floodplain Geomorphology
Source: ScientificWorldJournal. 2014 Jan 22;2014:309673. doi: 10.1155/2014/309673 (PMC3920808; doi:10.1155/2014/309673)
Supplement: Supplementary file 1 — A database of large dams in southeast Australia. ‘Large' is defined as having a catchment area greater than 100 km2, and a capacity capable of storing greater than 20% of average annual inflow. Some dams appear twice, indicating their original and enlarged capacities. The dams are ranked by catchment area. The ‘Purpose' column indicates the primary function(s) of the dams, using the key: (S) Storage for water supply; (I) Irrigation; (H) Hydropower; (F) Flood mitigation; (R) Stormwater retention; (P) Supply to coal-fired power stations. [file 309673.f1.pdf]

Dam Wall	Dam State	River	Purpose	Catchment Area km^2	Capacity ML	Surface Area Km^2	Height m	Dam Date
Menindee	NSW	Darling	I S	575000	1794000	45800	18	1960
Hume	NSW	Murray	I H	15300	3038000	20240	51	1936
Burrinnjuck	NSW	Murrumbidgee	I H	13000	1026000	5500	79	1956
Warragamba	NSW	Warragamba	S H	9000	1886000	7495	137	1960
Wyangala	NSW	Lachlan	I	8300	1220000	5300	85	1971
Wyangala	NSW	Lachlan	I	8300	374860	0	61	1935
Wivenhoe	QLD	Brisbane	SFRHH	7020	1150000	10800	59	1985
Keepit	NSW	Namoi	I H	5700	423000	4375	55	1960
Copeton	NSW	Gwydir	I S	5360	1364000	4600	113	1976
Boondooma	QLD	Boyne	S I P	4200	212000	1920	64	1983
Eildon	VIC	Goulburn	I H	3870	3390000	13832	79	1927
Dartmouth	VIC	Mitta Mitta	I H S	3611	4057000	6300	180	1979
Wuruma	QLD	Nogo	I	2320	194000	1780	46	1969
Eppalock	VIC	Campaspe	I S	2030	312000	3230	45	1962
Pindari	NSW	Severn	S.I	2000	37500	316	45	1969
Glenmaggie	VIC	Macalister	I	1890	190000	1760	37	1927
Jindabyne	NSW	Snowy	H	1850	688287	3034	72	1967
Coolmunda	QLD	Macintyre	I S	1760	75200	1740	20	1968
Bjelke-Petersen	QLD	Barker	I	1670	125000	2150	43	1988
Blowering	NSW	Tumut	I H	1600	1628000	4455	112	1968
Cairn Curran	VIC	Loddon	I S	1590	148000	1900	44	1956
Somerset	QLD	Stanley	SFRH	1330	369000	4350	50	1959
Fred Haigh	QLD	Kolan	I S	1310	586000	5340	52	1975
Glenbawn	NSW	Hunter	I F S	1300	750000	2700	100	1987
Glenbawn	NSW	Hunter	I F S	1300	360000	1700	78	1958
Glenlyon	QLD	Pike	I S	1295	254000	1800	62	1976
Teddington Wr	QLD	Tinana	S	1190	3880	140	7	1933
Talbingo	NSW	Tumut	H	1090	921400	1936	162	1971
Windemere	NSW	Cudgegong	I R	1070	368000	2030	67	1983
Buffalo	VIC	Buffalo	I S	1062	24000	341	30	1965
Googong	NSW	Queanbeyan	S	873	124500	680	62	1977
Tullaroop	VIC	Tullaroop	I S	742	74000	742	24	1959
Eucumbene	NSW	Eucumbene	H	673	4798400	14452	116	1958
Leslie	QLD	Sandy	I S R	603	47100	724	30	1965
Leslie	QLD	Sandy	I S R	603	108000	1260	33	1986
Tantangara	NSW	Murrumbidgee	H	471	254099	2118	45	1960
Borumba	QLD	Yabba	I S	465	42600	502	53	1964
Chaffey	NSW	Peel	I S	420	61800	542	54	1979
Brogo	NSW	Brogo	I	396	9000	102	43	1976
Nillahcootie	VIC	Broken	S I	389	40000	532	35	1967
Mount Bold	SA	Onkaparinga	S	388	45900	308	56	1963
Mount Bold	SA	Onkaparinga	S	388	30200	0	0	1937
North Pine	QLD	North Pine	S R	347	202000	2150	44	1976
Upper Yarra	VIC	Yarra	S	337	207200	750	89	1957
Blue Rock	VIC	Tanjil	S	337	200000	873	75	1984
Tumut	NSW	Tumut	H	332	52793	203	86	1958
Cressbrook	QLD	Cressbrook Cr	S	325	83000	530	63	1983
Nepean	NSW	Nepean	S	320	45930	357	81	1935
Malmsbury	VIC	Coliban	I S	290	18000	301	24	1870
Cania	QLD	Three Moon Cr	I A	280	89000	720	54	1982
Lostock	NSW	Paterson	I	280	20000	220	38	1971
Glennies Creek	NSW	Glennies Creek	I S	233	283000	1540	67	1983
Carcoar	NSW	Belubula	I	230	35800	385	58	1970
South Para	SA	South Para	S	228	51190	444	48	1960
South Para	SA	South Para	S	228	44900	0	0	1958
Moogerah	QLD	Reynolds	I S P	225	92500	878	40	1961
Lauriston	VIC	Coliban	I S	223	20000	208	33	1941
Hinze	QLD	Nerang	S	209	165000	972	62	1989
Hinze	QLD	Nerang	S	209	41820	486	44	1976
Corin	NSW	Cotter	S	197	74970	320	76	1968
Chichester	NSW	Chichester	S	197	17740	152	41	1923
Cooby Creek	QLD	Cooby Creek	S	184	20900	301	31	1942
Moondarra	VIC	Tyers	S	172	30400	243	41	1962
Pejar	NSW	Wollondilly	S	166	9000	140	26	1979
Tooma	NSW	Tooma	H	153	28124	0	67	1961
Avon	NSW	Avon	S	141	146700	1056	72	1935
Cataract	NSW	Cataract	S	130	94300	851	59	1907
Thomson	VIC	Thomson	S I	126	1123000	220	166	1985
Toolondo	VIC	Mt Talbot Creek	I S	126	106500	0	6	1954
Connolly	QLD	Rosenthal Cr	S I	124	2590	55	22	1927

Myponga	SA	Myponga	S	124	27130	280	50	1962
Moora Moora	VIC	Castle	S	124	6300	0	2	1934
Warren	SA	South Parra	S	119	4770	105	26	1916
Perseverance	QLD	Perseverance	S	117	30900	220	53	1965
Tarago	VIC	Tarago	S	109	37500	359	34	1968
Maroon	QLD	Burnett	I	106	38400	326	52	1974
Maroondah	VIC	Watts	S	104	28370	197	41	1927
Mangrove Cr	NSW	Mangrove Cr	S	104	170000	700	80	1982
Ginninderra	NSW	Ginninderra	R	100	3700	105	17	1975
